# Overview of IgG4 – Related Disease

**Published:** 2017

**Authors:** R Opriţă, B Opriţă, D Berceanu, IB Diaconescu

**Affiliations:** * Department of Gastroenterology, Floreasca Emergency Hospital, Bucharest, Romania

**Keywords:** IgG4- related disease, IgG4 molecule, diagnosis, histopathology, physiopathology, B cells, T cells, treatment, rituximab

## Abstract

Rationale (hypothesis): IgG4-related disease (IgG4-RD) is a pathological entity recently recognized by the medical world that can affect any organ or system. However, there is insufficient data about this disease in medical literature.

Aim (objective): A more extensive clarification of the IgG4 molecule, the diversified aspects of IgG4-related disease, and the response of this disease to treatment, will provide a crucial understanding of the immune system and other diseases now known to be associated with IgG4.

Methods and results: The MEDLINE online medical database was used, and, after a comprehensive review of medical articles regarding IgG4-RD, published after 2003, using the search words “IgG4- related disease” and “IgG4 molecule”, we have described the clinical, pathological and therapeutic features of IgG4-RD, as well as the presence of the IgG4 molecule in the evolution, diagnosis and management of this syndrome. We characterized the potential disease mechanisms and discussed early observations related to treatment.

Discussion: Given the response to immunosuppressive therapy, it is hypothesized that IgG4-related disease is most likely an autoimmune disease. Therefore, IgG4-related disease is a fibro-inflammatory condition that can affect any organ and can lead to the formation of pseudotumoral lesions requiring differential diagnosis with various malignancies. Positive diagnostic criteria are histopathological and require at least two features out of the following three: dense limphoplasmocitary infiltrate, storiform fibrosis, obliterative phlebitis.

## Introduction – history, definition, and diagnostic criteria

IgG4-related disease (IgG4-RD) is a pathological entity recently recognized by the medical world that can affect any organ or system. The basics of this condition have begun to be constructed since 2003, when patients with autoimmune pancreatitis have also been observed to have extrapancreatic manifestations [**1**]. Damage can be done to a single organ or to multiple tissues or systems that are involved in a synchronous or metachronous way. The clinical expression of the disease varies depending on the organs involved. The histopathological examination is considered the gold standard in obtaining a diagnosis, and the observed morphopathological changes are similar regardless of the affected organ. From this perspective, IgG4-related disease may be compared to sarcoidosis, another multisystemic disease with similar morphopathological changes in any sample of tissue that is involved. The histological criteria are: diffuse lymphoplasmacytic infiltrate, numerous IgG4 positive plasma cells in the examined tissue, storiform fibrosis (resembling the spokes of a cartwheel), eosinophils in mild to moderate quantities, obliterative phlebitis, and pseudotumoral lesions that tend to form in the affected organs [**2**]. Though initially considered that markedly elevated serum IgG4 levels were essential for diagnosis, it is known now that up to 30% of the patients may have a normal serum concentration despite the histopathological criteria supporting a positive diagnosis [**3**]. However, many aspects of the disease remain unclear.

## Epidemiology

Epidemiology of the disease is insufficiently described, but some demographic peculiarities stand out. Most patients are males over 50 years of age with a male/ female ratio of 3/ 1. Here, what should be noted is the discrepancy with other classic autoimmune diseases such as Sjogren's syndrome, systemic lupus erythematosus, primary biliary cirrhosis, which are predominantly found in females. The incidence and prevalence of the disease are not known, because only studies of autoimmune pancreatitis were conducted mainly in Japan. The prevalence is 0.8 cases/ 100,000 inhabitants/ year, accounting for 6% of the total cases of chronic pancreatitis [**4**].

## Pathological entities included in IgG4-RD

Type 1 autoimmune pancreatitis (AIP) is associated with an increased serum IgG4 level and it was the first disease included in the broad spectrum of IgG4- related disease. The assumption that IgG4-RD is a multisystemic disease was raised from the fact that patients with autoimmune pancreatitis also express extrapancreatic manifestations. Fundamentally, IgG4-RD can affect any organ: the pancreas, bile ducts, eyes, salivary glands, lungs, heart, kidneys, skin, aorta, ganglia, meninges, prostate, breast, thyroid, retroperitoneal tissue, etc. [**5**]. Therefore, over time, more and more diseases have been included in the spectrum of IgG4-related disease. Considered a variant of Sjogren's syndrome, Mikulicz syndrome consists in the swelling of the submandibular, lacrimal and parotid glands, and is now reclassified as being IgG4-related sialadenitis/ dacryoadenitis [**6**]. Kuttner tumor is an increase in the volume of submandibular glands in the context of a lithiasis or an infectious etiology, while bilateral damage to the submandibular glands in the absence of a precise etiology should be regarded as pertaining to IgG4-RD [**7**]. Also, Riedel thyroiditis is now a small part of the many diseases included in the IgG4-RD family. In the initial stages of the disease, the diagnosis is effortless based on classical histopathological criteria, but as the disease progresses and fibrotic tissue replaces the parenchyma, it is troublesome to obtain a definite diagnosis [**8**]. The affliction of the thoracic aorta in ~10% of the cases is found in IgG4-RD in the form of a chronic sclerosing aorta. The abdominal aorta (abdominal aortitis, often associated with retroperitoneal fibrosis), pancreas - type 1 autoimmune pancreatitis, biliary tract (10% of the patients with primary sclerosing cholangitis are eventually diagnosed with IgG4-RD) may also be involved. Ocular and periorbital tissue may be affected, but the reported frequency appears to be very low due to refraining from performing a biopsy. Eosinophilic angiocentric fibrosis causes pseudotumoral lesions of the eye or upper respiratory tract.

## Histopathology – central role in diagnosis

Although it has a high concentration in the serum, IgG4 immunoglobulin does not appear to play a central role in the pathophysiology of the disease [**9**]. In contrast, for pemphigus vulgaris or idiopathic membranous nephropathy, IgG4 triggers immune-mediated processes that lead to tissue destruction. The histopathological examination performed in these diseases differs substantially from the changes observed in IgG4. The main element is the lymphoplasmacytic infiltrate observed in most cases, with the acknowledgment that in advanced cases of retroperitoneal fibrosis or Riedel thyroiditis, extensive fibrosis virtually replaces the entire parenchyma, and typical changes that support the diagnosis of IgG4 are not found. Lymphocytes are especially CD4-positive T lymphocytes, and the B cells are confined to small cohesive lymphoid aggregates [**10**]. Eosinophils do not dominate the histopathological picture but justify the patient's allergic history. Many patients have a history of atopy, eczema, asthma, or chronic sinusitis. Besides the abundant cellularity of lymphocytes, plasmocytes and eosinophils, the storiform fibrosis is characterized by short, interlaced, curved collagen beads arranged in a matted and irregularly whorled pattern, which originate from a central point and radiate towards the periphery. Obliterative phlebitis is seen as veins with narrow lumens (sometimes completely stenotic) by the lymphoplasmacytic infiltrate; this process is always found in pancreatic and submandibular salivary glands, but less frequently in the lacrimal glands [**2**]. The differential diagnosis with a vasculitis is based on the absence of neutrophils, fibrinoid necrosis, or nuclear dust. Histopathological elements that make a diagnosis of IgG4-RD improbable are epithelioid cell granulomas, neutrophil infiltration, giant cells, and necrotic zones [**11**].

Most of the plasmocytes are IgG4 positive, a change that can also be found in chronic inflammatory processes. This finding is insufficient to support the diagnosis and should be accompanied by other histopathological findings. A minimal percentage of IgG4-positive plasmocytes for diagnosis has not yet been established, but 30 plasmocytes per high-powered field or an IgG4: total IgG ratio of greater than 40% are suggestive for the diagnosis of IgG4-related disease. A lower cutoff point for IgG4-positive cells is accepted in patients with suggestive pathological findings [**12**].

## IgG4 molecule

Patients with IgG4-RD may have a polyclonal increase in immunoglobulins regarding the IgG4 subclass, a feature that can be useful in reinforcing the diagnosis but is not required to be present or self-sufficient for the diagnosis. A high serum concentration of IgG4 immunoglobulins is found in patients with multiple organ damage. A wrong diagnosis of IgG4-RD is commonly encountered because overestimation is given to the role of serum or tissue IgG4 (up to 30% of the patients may have normal IgG4 values) [**3**]. For type 1 autoimmune pancreatitis, the sensitivity of serum IgG4 increases depending on the stage of the disease; it should be stated that some studies included patients with type 2 autoimmune pancreatitis with different characteristics at a microscopic level (neutrophil infiltrate, epithelioid granulomas) [**13**]. No other serum markers have an established role in the diagnosis.

## Clinical presentation and differential diagnosis

Generally, patients with IgG4-RD have a subacute clinical presentation. A minority (less than 10% of the total) presents with acute symptoms such as fever, weight loss, an increase in acute phase reactants and other manifestations of a systemic inflammatory process. Frequently, the patient presentation is that of single organ damage.

Patients with type 1 autoimmune pancreatitis have a clinical presentation similar to that of patients with pancreatic cancer: obstructive jaundice, weight loss, mild abdominal pain. Therefore, the diagnosis was obtained laparoscopically until recently, but with a better understanding of the IgG4-related disease concerning specific imaging changes (diffusely swollen, sausage-shaped pancreas, lymphadenopathy) a high index of suspicion should be made. A differential diagnosis with primary sclerosing cholangitis is difficult based only on imaging studies and is based on histopathological changes [**14**].

Patients with type 1 autoimmune pancreatitis can have liver damage that includes portal inflammation with or without perihepatitis, obstruction of large bile ducts, portal fibrosis, lobular hepatitis, canalicular cholestasis. The differential diagnosis of autoimmune hepatitis is based on autoantibodies (ANA - antinuclear antibodies, ASMA-anti-smooth muscle antibodies, p-ANCA - perinuclear anti-neutrophil cytoplasmic antibodies and/ or anti-SLA/LP-specific antibodies against soluble liver antigen/ liver-pancreas). The gallbladder localization of the disease appears as acalculous cholecystitis, but the discovery is often made after the surgical removal, microscopic examination and staining for IgG4 [**15**].

The orbit is a frequently omitted location of IgG4-RD, often misclassified as idiopathic orbital inflammation. Structures that may be affected are the lacrimal gland, lacrimal ducts, and retrobulbar zone. A differential diagnosis is required with neoplasms including lymphomas and pseudotumoral inflammatory lesions caused by granulomatosis with polyangiitis.

Sialadenitis of IgG4-RD mainly affects the submandibular gland bilaterally unlike Sjogren's syndrome that never affects only the submandibular gland. The primary differential diagnosis is with Sjogren's syndrome that preferentially affects the parotid gland (more rarely affected in IgG4-RD). Minor salivary glands can be affected at a microscopic level without a macroscopic change.

The IgG4-related disease can affect the thoracic or abdominal aorta in the form of aortitis. Inflammatory abdominal aortitis can lead to the formation of aneurysms and aortic dissections, as well as retroperitoneal fibrosis as the disease progresses. Retroperitoneal fibrosis may or may not be included in IgG4-RD; the initial stages are characterized by an abundant lymphoplasmacytic infiltrate that is replaced in time with fibrous tissue. It is difficult to establish whether an advanced stage of retroperitoneal fibrosis belongs to IgG4-RD or other pathological entities.

The IgG4-related lung disease site comprises the following symptoms: cough, haemoptysis, shortness of breath, pleural effusion, chest pain. Interstitial pneumonia or pseudotumoral inflammatory lesions complete the clinical spectrum. Histologically and radiologically, four types of pulmonary patterns are described: nodular, bronchovascular, interstitial, round ground-glass opacities. Renal disease in IgG4 disease is difficult to distinguish from renal carcinoma. The microscopic examination reveals tubulointerstitial nephritis with diffuse or sometimes localized lymphoplasmacytic infiltration with associated interstitial fibrosis. Clinical features are hematuria, proteinuria, renal failure and other extrarenal manifestations in an overwhelming proportion of cases [**16**]. Other organs involved can be the pituitary gland, meninges, skin, prostate, pericardium, lymph nodes. Ganglia involvement can be isolated (adjacent to another parenchymal lesion) or generalized; it is important to mention that a lymph node biopsy does not reveal storiform fibrosis commonly found in other locations, and a diagnosis of reactive follicular hyperplasia is usually made, as other diagnoses such as IgG4-RD are not considered. The frequency of localization in various organs is not fully known, as many lesions are asymptomatic such as cutaneous erythematous papular eruptions.

## Pathophysiology

The pathophysiology of the disease is complex and should start with the description of the IgG4 molecule. IgG4 has a concentration of less than 5% of total IgG immunoglobulins, being the subclass with the most stable interindividual concentration. IgG4 does not activate the classical complement pathway and plays a limited role in the immune process. The production is predominantly controlled by T helper type 2 lymphocytes through secretion of cytokines - interleukins 4 and 13 [**17**]. The molecule plays a pivotal role in the pathogenesis of diseases such as bullous pemphigus by interacting with desmoglein-1 [**18**]. Also, in podocytes, the interaction of IgG4 with M-type phospholipase A2 explains the appearance of idiopathic membranous glomerulonephritis. Thrombotic thrombocytopenic purpura is initiated by binding of IgG4 with ADAMTS13 (a disintegrin and metalloproteinase) [**19**]. The definitive role of IgG4 in the disease remains to be established, whether this subclass plays a central role in the pathogenesis of the disease, or is an epiphenomenon in other processes without a direct implication in the pathophysiology of the disease.

Is essential to distinguish IgG4-RD from a lymphoma. Type B lymphomas have a predominantly lymphocytic infiltrate of type B lymphocytes, unlike IgG4-RD where lymphocytes are predominantly T-type. Additional clonal studies are always required for a correct diagnosis. Inflammatory infiltrations adjacent to neoplastic processes are comprised of focal IgG4 positive plasmocytes aggregates, as opposed to their diffuse layout in IgG4-related disease. There is a similarity in the structure of human carbonic anhydrase type II and that secreted by Helicobacter pylori (alpha carbonic anhydrase). Patients with autoimmune pancreatitis frequently have antibodies against Helicobacter pylori; thus, these antibodies directed against infectious agents may act as autoantibodies directed against various tissue structures in patients with a genetic predisposition [**20**]. IgG4-RD is regarded as both an allergic as well as an autoimmune disorder.

The role of type T and B-lymphocytes is still not fully understood. Finding a CD 4+ cytotoxic T cell remains the main objective in unveiling the main part of the pathophysiology, bearing in mind that these cells are found in the highest concentration within the lesions. This cell population secretes molecules such as interleukine-1, TGF-β or IFN-γ, which play a major role in the fibrosis that characterizes the late stages of the disease. As well, is important to mention that the CD4+ cytotoxic T cells have a molecule on their surface, called SLAMF7, not formerly labeled on T cells, only B cells [**21**].

An assumption is made that the CD 4+ cytotoxic T cells are continuously stimulated by antigen presentation performed by B cells or plasmablasts. A reduction in B cell concentration is not accompanied by a normal level of IgG4 serum level even though the patient is in clinical remission, implying that the production of IgG4 is still assured by long-lived plasma cells.

## Treatment

There is contradictory information about a periodic monitoring of serum IgG4 levels to assess the response to treatment. Although there is a decrease in IgG4 concentration after the start of corticosteroid treatment, serum levels remain high in most patients (in a study in Japan, about 63% of the patients had an elevated IgG4 level after treatment, despite some patients entering clinical remission). Only 30% of the cases with persistent elevated IgG4 levels had relapses. Also, 10% of the patients with normal IgG4 levels also experienced recurrences [**22**]. One study showed that the prozone effect led to false negative results of IgG4 concentration. The prozone effect is more common in those with active disease, and that is, precisely in patients in whom a correct and rapid diagnosis is crucial to begin the treatment. The concentration of IgG4 is measured by nephelometry; it is carried out by measuring the turbidity in a water sample by passing light through the sample being measured. The amount of light passing through is dependent on the level of aggregates formed by the interaction of the antibodies present in the reagent to the antigens (IgG4) present in the patient sample. If the antigen is present in the solution, the light scatter signal begins to decrease, a phenomenon known as the prozone effect. The prozone effect can be bypassed with the dilution of the sample as to obtain and maintain an antibody level greater than that of the antigen [**23**].

Treatment should be instituted urgently when vital organs are affected, as the disease can evolve to severe organ dysfunction. Not all disorders require immediate treatment. Lymphadenopathy in the disease is often asymptomatic and may persist for decades without clinical consequences. Glucocorticoids are the first line of treatment. The starting dose should be 0.6 mg/ kg of prednisolone for 2-4 weeks, then adjusted to 5 mg/ day over a period of 3-6 months. The treatment can be continued for up to 3 years with a dose of 2.5-5 mg/ day [**24**]. Other recommendations suggest the treatment should be stopped after 3 months [**22**]. Corticosteroid therapy is effective but relapses frequently occur. Methotrexate, azathioprine, or mycophenolate mofetil are other agents that can be used but have not been included in clinical trials to study their effectiveness. In patients with a recurrent or refractory disease, rituximab may be used with spectacular results. When rituximab is administered, there is a marked decrease in IgG4 level, with stable concentrations of the other immunoglobulin subclasses, and with clinical remission registered within a few weeks. The effect of rituximab is obtained by lowering the concentration of CD 20 positive B cells that differentiate into IgG4 producing plasmocytes [**25**]. Patients with longstanding disease, in whom extensive fibrosis has virtually replaced the parenchyma of the affected organ, have a lower rate of response to the glucocorticoid or rituximab treatment.

## Conclusions

Given the response to immunosuppressive therapy, it is hypothesized that IgG4-related disease is most likely an autoimmune disease. Therefore, the IgG4-related disease is a fibro-inflammatory condition that can affect any organ and can lead to the formation of pseudotumoral lesions requiring differential diagnosis with various malignancies. Positive diagnostic criteria are histopathological and require at least two features out of the following three: dense lymphoplasmacytic infiltrate, storiform fibrosis, obliterative phlebitis. IgG4 immunoglobulins weakly bind the complement and are considered to have anti-inflammatory properties. Various autoantibodies have been described in the disease, but their contribution to lesion progression remains to be established. The genetic substrate needs to be properly assessed by clinical studies as well as the possible role of bacteria in pathogenesis through human microbiota studies. Also, it is necessary to identify the target antigens of IgG4 as well as the role of fibroblasts and macrophages in the pathogenesis of the disease. A more thorough insight of the IgG4 immunoglobulin, the distinct aspects of the IgG4-related disease, and the response of this syndrome to treatment, especially B-cell reduction, may return critical understandings of the immune system and other conditions now known to be associated with IgG4.

## Conflict of interest

None declared.
